# The photosensitizer DH-I-180-3 regulates intracellular bacterial growth by increasing the secretion of proinflammatory cytokines via the NF-κB- and MAPK-mediated signaling pathways and promoting phagosome maturation in *Salmonella*-infected mouse macrophages

**DOI:** 10.71150/jm.2502003

**Published:** 2025-06-04

**Authors:** Hyo-Jung Kim, Eui-Kwon Jeong, Hyo-Ji Lee, Yu-Jin Jung

**Affiliations:** 1 Department of BIT Medical Convergence Graduate Program, Kangwon National University, Chuncheon 24341, Republic of Korea; 2 Department of Biological Sciences, Kangwon National University, Chuncheon 24341, Republic of Korea; 3 Kangwon Radiation Convergence Research Support Center, Kangwon National University, Chuncheon 24341, Republic of Korea

**Keywords:** bactericidal activity, DH-I-180-3, inflammatory signaling pathway, phagosome maturation, photodynamic therapy, photosensitizer, reactive oxygen species

## Abstract

Photodynamic therapy (PDT) is a known strategy for treating cancer; in PDT, photosensitizers are activated by light stimulation and then induce reactive oxygen species (ROS) production to damage cancer tissues. Recently evidence has shown that PDT can also be used as a novel treatment strategy to control pathogenic bacteria. In previous studies, the photosensitizer DH-I-180-3 was reported to effectively regulate multidrug-resistant *Mycobacterium tuberculosis* growth. Here, we confirmed the effects of DH-I-180-3 on the antibacterial activity and inflammatory response of macrophages to *Salmonella*. Photoactivated DH-I-180-3 regulated intracellular bacterial growth in *Salmonella*-infected macrophages. Moreover, DH-I-180-3 increased intracellular ROS levels in *Salmonella*-infected macrophages. The phosphorylation of the intracellular signaling proteins IκBα and JNK1/2 was increased in DH-I-180-3-treated *Salmonella*-infected macrophages. Additionally, we observed that DH-I-180-3 significantly increased the mRNA expression and protein secretion of the proinflammatory cytokine TNF-α and promoted phagosome maturation by upregulating EEA1, LAMP1, and Cathepsin D in *Salmonella*-infected macrophages. Overall, these results demonstrate that photoactivated DH-I-180-3 enhances the bactericidal response to intracellular bacterial infection by promoting inflammatory signaling pathways and phagosome maturation. Therefore, DH-I-180-3 has the potential to be developed into PDT for treating bacterial-infection.

## Introduction

Photodynamic therapy (PDT) is a cancer treatment technology that uses photosensitizers to induce reactive oxygen species (ROS)-mediated tumor cell death (Li et al., 2020a). When photosensitizers are exposed to light of a specific wavelength, they become excited from the ground state and induce ROS production through type I and II mechanisms (Shen et al., 2021). An ideal photosensitizer should have a strong absorption peak in the red to near-infrared spectral region (between 650 and 800 nm) and a substantial triplet quantum yield while being a single pure compound with lower manufacturing costs and better stability (Abrahamse and Hamblin, 2016). In addition, the use of PDT as an antimicrobial PDT (aPDT) is constantly being studied to overcome the problem of antibiotic-resistance among pathogens (Polat and Kang, 2021; Youf et al., 2021). The most common PS employed in aPDT include porphyrins, the phenothiazines toluidine blue O (TBO) and methylene blue (MB), aminolevulinic acid (ALA) and methyl-aminolevulinate (MAL), psoralen, riboflavin, chlorophyll a, and curcumin (Almenara-Blasco et al., 2024; Youf et al., 2021). Among pathogens, Gram-negative bacteria are relatively difficult to treat with aPDT because their membrane barrier structure prevents their absorption of photosensitizers (Sperandio et al., 2013). Therefore, a different approach is needed to effectively control Gram-negative bacteria via PDT.

The cascade of inflammatory signaling pathways is initiated by innate immune receptors, such as pattern recognition receptors (PRRs), that recognize common harmful stimuli (Ashley et al., 2012). Toll-like receptor 4 (TLR4), which is a representative PRR, recognizes the lipopolysaccharides (LPSs) of Gram-negative bacteria and triggers the activation of the downstream nuclear factor-κB (NF-κB) and mitogen-associated protein kinase (MAPK) signaling pathways. Activation of these signaling cascades results in phenomena such as the production and release of cytokines, chemokines, and growth factors and thus contributes to the inflammatory response (Li and Wu, 2021). The ROS that are generated during PDT induce an acute inflammatory response at the target site by damaging and destroying tumor tissues (Falk-Mahapatra and Gollnick, 2020; Zheng et al., 2016). However, the mechanism underlying the immune-stimulating effect of PDT is not fully understood, and further research in microbial infection models is still needed (Reginato et al., 2014).

DH-I-180-3 is a novel derivative of chlorin that has a tetrapyrrole structure and a maximum absorption wavelength of 666 nm. In a previous study, DH-I-180-3 treatment delayed tumor growth and improved survival rates in mice compared to treatment with the conventional photosensitizer Photofrin (Lim et al., 2003). In addition, DH-I-180-3 has been shown to effectively control multidrug-resistant *Mycobacterium tuberculosis* following photoactivation (Sung et al., 2013). In these studies, DH-I-180-3-mediated PDT has been demonstrated to exert excellent antitumor and antituberculosis effects. However, the effects of DH-I-180-3 on the antibacterial responses to Gram-negative bacteria and the immune stimulation of bacterium-infected cells have not been elucidated. *Salmonella enterica* is a Gram-negative bacterium composed of over 2500 serotypes. Serotypes are divided based on the antigenic variability of O-antigens in LPS (Coburn et al., 2007). Among them, *Salmonella enterica* serovar Typhimurium (*S.* Typhimurium) is the most common cause of gastroenteritis worldwide and a foodborne pathogen (Nilsson et al., 2019). The LPS, flagellin, and CpG-DNA of *S.* Typhimurium are known to act as pathogen-associated molecular patterns (PAMPs), stimulating TLRs (Gilchrist et al., 2015). Therefore, in this study, experiments were conducted to investigate the effects of DH-I-180-3 on *S.* Typhimurium and its immune-stimulating effects on macrophages during *S.* Typhimurium infection.

## Materials and Methods

### Reagents

DH-I-180-3 was provided by Professor Chang Hee Lee (Kangwon University, Korea) and was dissolved in dimethyl sulfoxide (DMSO). All the following antibodies that were used for western blotting were purchased from Cell Signaling Technology (CST, USA); anti-p-IκBα, anti-IκBα, anti-p-p38, anti-p-JNK, anti-p-ERK1/2, anti-ERK1/2, anti-Rab5A, anti-Rab7, anti-EEA1, anti-LAMP1, anti-Cathepsin D, and anti-β-actin antibodies.

### Cell culture

The mouse macrophage cell line, RAW264.7, was purchased from the American Type Culture Collection (ATCC, USA). RAW264.7 cells were maintained in DMEM supplemented with 10% fetal bovine serum and 1% penicillin/streptomycin at 37°C and 5% CO_2_.

### Bacterial strains and culture conditions

*S.* Typhimurium strain SL1344 was inoculated from a frozen stock onto LB agar and incubated at 37°C for 24 h. A single colony was cultured in LB broth at 37°C with shaking (160 rpm). Under these conditions, an optical density of 1 corresponded to a bacterial count of 1 × 10^8^ colony forming units (CFU).

### *S.* Typhimurium infection in vitro

*S.* Typhimurium infection was performed as previously described (Lee et al., 2020a). RAW264.7 cells were seeded at 2 × 10^4^ cells/well and incubated for 2 days at 37°C and 5% CO_2_. The cells were washed with PBS and the medium was then replaced with medium supplemented with *S.* Typhimurium at a multiplicity of infection (MOI) of 5. The plate was centrifuged at 1,000 rpm for 10 min and then incubated at 37°C and 5% CO_2_ for 30 min. After 30 min, the cells were washed with PBS and then treated with 0.1 μg/ml DH-I-180-3 or DMSO in the presence of 60 μg/ml gentamicin, which removed extracellular bacteria. After 1 h, the cells were irradiated with a halogen lamp at 666 nm for 1 min.

### Intracellular CFU assay

The intracellular CFU assay was performed as previously described (Lee et al., 2021). RAW264.7 cells were infected as described above. After 4 h of light irradiation, the *S.* Typhimurium-infected cells were lysed with 0.1% Triton X-100. The appropriate number of bacteria was subsequently spread on LB agar by serial dilution and incubated for 24 h at 37°C. The number of intracellular bacteria was determined by calculating the number of cultured CFUs.

### Cell viability assay

To evaluate the cell viability of RAW264.7 cells treated with different doses of DH-I-180-3, cells were treated with 0.1, 1, or 10 μg/ml DH-I-180-3 and irradiated with a halogen lamp at 666 nm for 1 min. After 72 h, the number of cells was counted using a hemocytometer following the trypan blue dye exclusion method. To evaluate the effect of DH-I-180-3 on the viability of *S.* Typhimurium-infected RAW264.7 cell viability, RAW264.7 cells were infected and treated with DH-I-180-3 as described above. After 48 h of light irradiation, the cell count was determined using the same method described above.

### Antibacterial experiments

To evaluate whether DH-I-180-3 exerts direct antibacterial effects on *S.* Typhimurium, a single colony was inoculated in LB broth and precultured for 30 min. Then, the cells were treated with 0.1 μg/ml DH-I-180-3 or DMSO and cultured for 1 h, followed by light irradiation for 1 min. After 4 h, the bacteria were inoculated on LB agar to determine the bacterial CFU.

### Measurement of intracellular ROS production

Intracellular ROS levels were measured as previously described via the use of 2',7'-dichlorofluorescein diacetate (DCF-DA; Sigma-Aldrich, USA), which reacts with intracellular ROS (Lee et al., 2020b). Briefly, RAW264.7 cells were seeded on cover glass and treated with 0.1 μg/ml DH-I-180-3 for 30 min before infection with *S.* Typhimurium at an MOI of 5. During infection, the cells were irradiated with light for 1 min to induce DH-I-180-3 photoactivation, followed by incubation at 37°C and 5% CO_2_ for 10 min. Then, the cells were treated with DCF-DA for 20 min and fixed with 4% paraformaldehyde. After the fixed cells were stained with DAPI, the cover glass was mounted with mounting solution. To observe the intracellular ROS levels, images of cells emitting DCF fluorescence were captured by confocal microscopy (Olympus, Japan). Then, 6–8 fields were randomly selected, and intracellular ROS production was quantified using ImageJ software. DCF fluorescence levels were analyzed by normalization to the DCF fluorescence intensity in control cells.

### Western blotting analysis

Western blotting analysis was performed as previously described (Woo et al., 2018). Briefly, RAW264.7 cells were treated with 0.1 μg/ml DH-I-180-3 for 30 min and infected with *S.* Typhimurium at an MOI of 5, followed by exposure to light for 1 min. Then, the cells were harvested at the indicated times and lysed with RIPA buffer supplemented with a protease inhibitor cocktail. The lysates were subsequently centrifuged at 14,000 rpm for 40 min at 4°C to separate the proteins. The protein concentrations in the lysates were determined using a Bradford assay (Bio-Rad, USA). Equal amounts of protein were separated using 10% SDS-PAGE and transferred to PVDF membranes (Millipore, USA). The membranes were incubated with primary and secondary antibodies after being blocked with skim milk. The target proteins were detected via an ECL detection system (Advansta, USA).

### Total RNA isolation and reverse transcription-polymerase chain reaction (RT-PCR)

*S.* Typhimurium-infected RAW264.7 cells were treated with DH-I-180-3 and irradiated with light as described above. The cells were harvested at the indicated time points, and total RNA was extracted with the RNeasy Mini Kit (Qiagen, Netherlands) according to the manufacturer's instructions. cDNA synthesis and PCR were performed as previously described (Lee et al., 2015). The oligonucleotide primers that were used were as follows: 5′-TTCTGTCTACTGAACTTCGGGGTAATCGGTCC-3′ (forward) and 5′-GTATGAGATAGCAATCGGCTGACGGTGTGGG-3′ (reverse) for tumor necrosis factor-alpha (TNF-α), 5′- CTGCTATGCTGCCTGCTCTTAC-3′ (forward) and 5′-GTAGACACCTTGGTCTTGGAGC-3′ (reverse) for interleukin 10 (IL-10), and 5′-AGGCTGTGCTGTCCCTGTATGC-3′ (forward) and 5′-ACCCAAGAAGGAAGGCTGGAAA-3′ (reverse) for β-actin. The amplified PCR products were analyzed in a 1.5% agarose gel and visualized under UV light after staining with ethidium bromide.

### Enzyme-linked immunosorbent assay (ELISA)

RAW264.7 cells were infected with *S.* Typhimurium as described above, and cell-free supernatants were harvested at 4 and 24 h after light irradiation. The levels of secreted TNF-α and IL-10 were measured with the appropriate kits (PeproTech; Thermo Fisher Scientific, Inc., USA) according to the manufacturer's instructions.

### Immunofluorescence staining

Immunofluorescence assays were performed as previously described (Lee et al., 2020a). Briefly, RAW264.7 cells were seeded on coverslips, treated with 0.1 μg/ml DH-I-180-3 for 30 min, infected with *S.* Typhimurium at an MOI of 5, and exposed to light for 1 min. Thirty min or 2 h after infection, cells were fixed with 4% paraformaldehyde and permeabilized with 0.2% Triton X-100, and then, the cells were incubated with mouse anti-*Salmonella* LPS and anti-mouse FITC (Invitrogen Thermo Fisher Scientific, Inc., USA) antibodies. To detect markers of phagosome maturation, the cells were incubated with anti-EEA1-Alexa Fluor^®^ 647 or anti-LAMP1-Alexa Fluor^®^ 647 (Novus Biologicals; Bio-Techne Corporation, USA) antibodies. The coverslips were stained with DAPI, mounted onto slides with mounting medium, and observed by using confocal microscopy (Nikon, Japan). Image analysis was performed using ImageJ software.

### Statistical analysis

All the experiments were performed with three independent samples and were repeated three times. Statistical analysis was performed using GraphPad Prism 5 software. The significance of differences between groups was analyzed with Student’s t test and one-way ANOVA followed by Tukey’s post hoc test for multiple comparisons. A p value < 0.05 (*p < 0.05; **p < 0.01; ***p < 0.001; and ns, not significant [p > 0.05]) was considered to indicate a significant difference.

## Results

### Photoactivation of DH-I-180-3 regulates intracellular bacterial growth in *S.* Typhimurium-infected macrophages

**Fig. 1.**
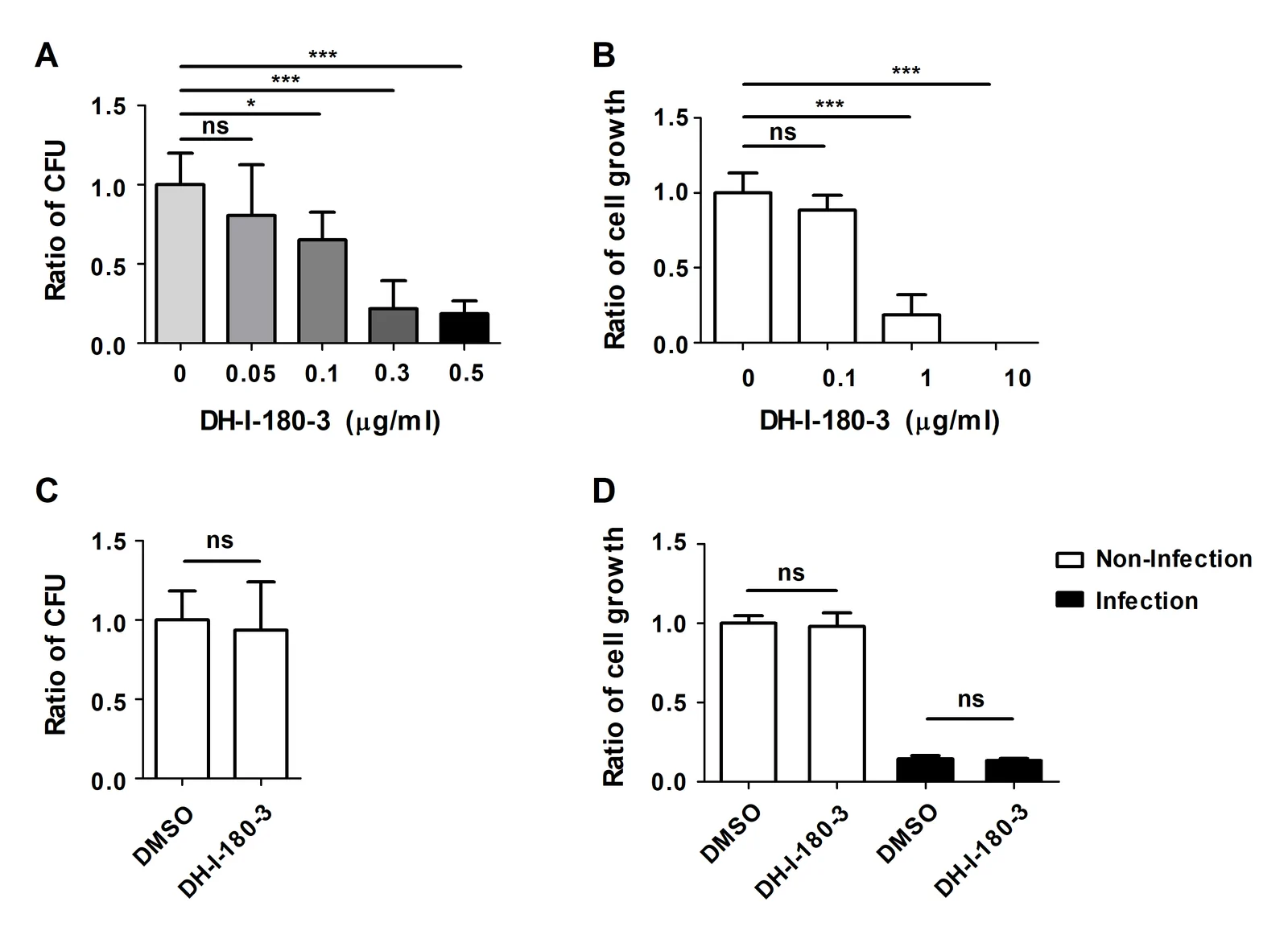
DH-I-180-3 regulates the growth of intracellular bacteria in *S.* Typhimurium-infected macrophages. (A) RAW264.7 cells were infected with *S.* Typhimurium and treated with different doses of DH-I-180-3. After 1 h, the samples were irradiated with light for 1 min to activate DH-I-180-3. After 4 h, the number of CFUs of intracellular bacteria was determined. (B) RAW264.7 cells were treated with different doses of DH-I-180-3 and irradiated with light. Trypan blue exclusion assays revealed cell growth after 72 h of DH-I-180-3 treatment. (C) *S.* Typhimurium was cultured medium supplemented with photoactivated DH-I-180-3 for 4 h. Bacterial growth was measured by counting the number of CFUs on agar plates. (D) *S.* Typhimurium-infected RAW264.7 cells were treated with DH-I-180-3 and then irradiated with light. After 48 h, cell viability was determined using the trypan blue exclusion assay. (A–C) The data were normalized to those of the nontreated control condition. (D) The data were normalized to those of noninfected and untreated cells. The data are expressed as the mean ± SD (n = 3).

To determine the effects of the photosensitizer DH-I-180-3 on *Salmonella*-infected macrophages, we infected RAW264.7 mouse macrophages with *S.* Typhimurium and then treated the cells with DH-I-180-3. Intracellular bacterial growth was evaluated under photodynamic conditions following irradiation with light at a wavelength of 666 nm. Four hour after light irradiation, the number of intracellular bacteria in DH-I-180-3-treated cells decreased in a DH-I-180-3 dose-dependent manner, as shown by the intracellular CFU assay (Fig. 1A). Since DH-I-180-3 alone exerted cytotoxic effects when administered at doses of 1 μg/ml or higher (Fig. 1B), a DH-I-180-3 concentration of 0.1 μg/ml was used for subsequent experiments.

Next, we confirmed whether DH-I-180-3 directly affects the growth of bacteria or infected host macrophages. The direct addition of DH-I-180-3 to *S.* Typhimurium growth medium and the induction of photoactivation did not inhibit bacterial growth (Fig. 1C). Additionally, photoactivation of DH-I-180-3 did not affect the viability of *S.* Typhimurium-infected RAW264.7 cells (Fig. 1D). These data indicate that DH-I-180-3 contributes to the regulation of intracellular bacteria in *Salmonella*-infected macrophages. However, since DH-I-180-3 does not exert direct antibacterial effects on *S.* Typhimurium, we focused on determining the effect of DH-I-180-3 on host cells.

### DH-I-180-3 treatment increases intracellular ROS production

**Fig. 2.**
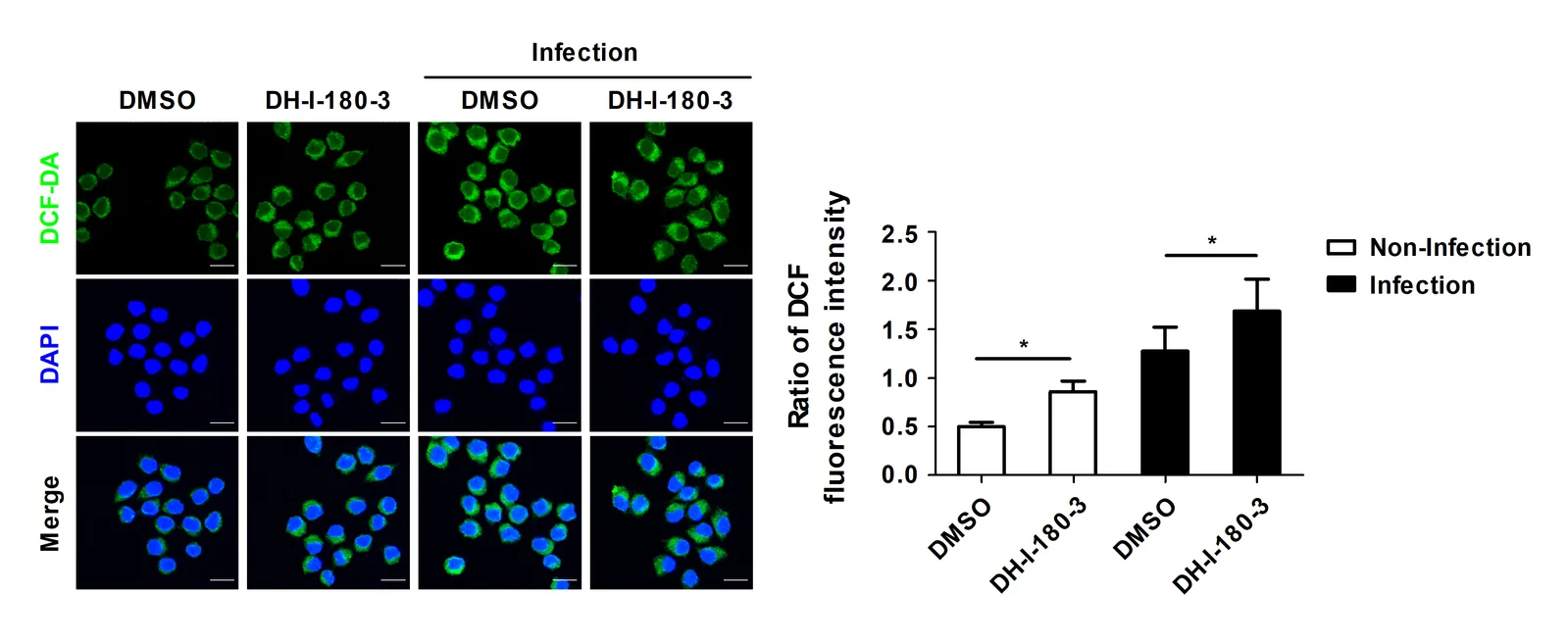
Photoactivation of DH-I-180-3 promotes intracellular ROS production. RAW264.7 cells were pretreated with DH-I-180-3, infected with *S.* Typhimurium and irradiated with light. Intracellular ROS levels were measured by confocal microscopic analysis of DCF-DA levels. The bar graph represents the relative intracellular ROS levels calculated according to the green fluorescence intensity. Scale bar = 10 μm. The data are expressed as the mean ± SD (n = 3 independent experiments with 50 cells per condition).

Pheophorbide a, which is a precursor of DH-I-180-3, reportedly increases intracellular ROS production in various types of cells (Choi et al., 2014; Kim et al., 2016). We determined whether the regulation of intracellular bacterial growth by DH-I-180-3 is associated with intracellular ROS levels. Compared with the control, treatment with DH-I-180-3 increased the intracellular ROS levels in the *S.* Typhimurium-infected RAW264.7 cells (Fig. 2). In addition, the DCF fluorescence intensity in noninfected cells was approximately 1.7-fold greater in DH-I-180-3-treated macrophages than in the control macrophages (Fig. 2). This finding suggests that DH-I-180-3 has activity as a photosensitizer. These results demonstrate that DH-I-180-3-induced ROS production in *Salmonella*-infected macrophages is associated with the regulatory effects of DH-I-180-3 on intracellular bacterial growth.

### Photoactivation of DH-I-180-3 increases NF-κB and MAPK signaling pathways activation in *S.* Typhimurium-infected macrophages

**Fig. 3.**
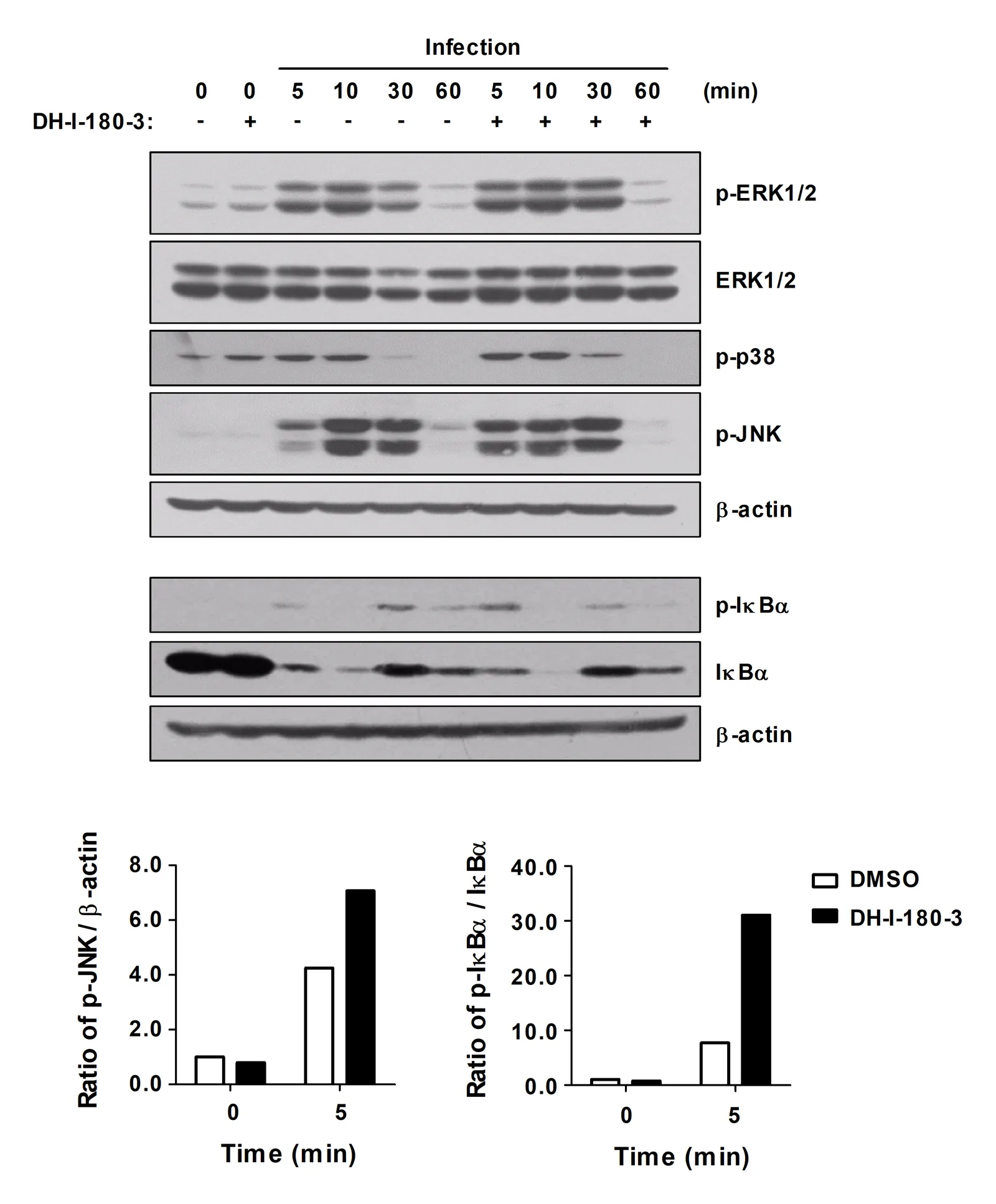
DH-I-180-3 accelerated NF-κB and MAPK signaling activation in *S.* Typhimurium-infected macrophages. RAW264.7 cells were pretreated with DH-I-180-3, infected with *S.* Typhimurium and irradiated with light. The phosphorylated and total protein levels of proteins related to the NF-κB and MAPK signaling pathways were measured by western blotting. β-Actin was used as a loading control. The bar graph shows the densitometric quantification of the bands corresponding to p-JNK/β-actin and p-IκBα/IκBα 5 min after *S.* Typhimurium infection. The data were normalized to those of noninfected and untreated cells. One representative result of three independent experiments is shown.

Intracellular ROS are pleiotropic physiological signaling agents that regulate cell growth, differentiation, and apoptosis (Sies and Jones, 2020). Signaling pathways that are related to inflammatory responses, such as the NF-κB, Jak/STAT, and STING pathways, are targeted by ROS in macrophages (Canton et al., 2021). To investigate the effects of DH-I-180-3, which induces intracellular ROS production, on inflammatory signaling pathways in *S.* Typhimurium-infected RAW264.7 cells, we assessed NF-κB and MAPK signaling pathways activation. Compared with those in control macrophages, the phosphorylation of ERK1/2, JNK, p38, and IκBα in infected macrophages was increased after DH-I-180-3 treatment and photoactivation (Fig. 3). In particular, our results revealed a significant increase in the levels of phosphorylated JNK and IκBα in the early stage of infection (5 min). These results suggest that DH-I-180-3 contributes to the regulation of intracellular bacteria in host cells by activating the NF-κB and MAPK signaling pathways immediately after *Salmonella* infection.

### DH-I-180-3 upregulates the mRNA and protein expression of TNF-α in *S.* Typhimurium-infected macrophages

**Fig. 4.**
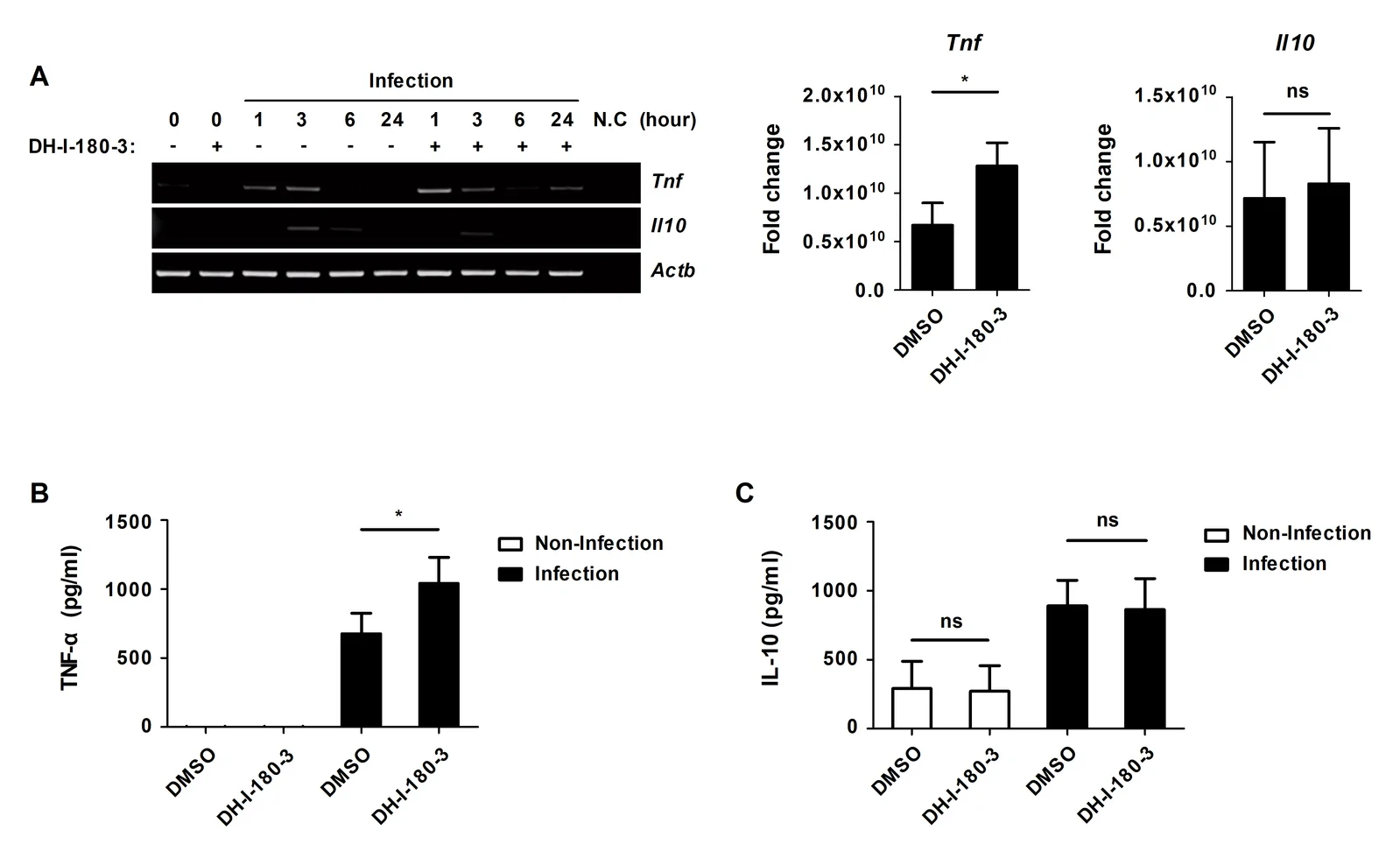
DH-I-180-3 increased the mRNA expression and protein secretion of the proinflammatory cytokine TNF-α in *S.* Typhimurium-infected macrophages. (A–C) RAW264.7 cells were infected with *S.* Typhimurium were treated with DH-I-180-3, and then irradiated with light. (A) mRNA expression of TNF-α and IL-10 was measured by RT-PCR analysis at the indicated time points. *Actb* was used as the loading control. The bar graphs represent the densitometric quantification of bands corresponding to *Tnf*/*Actb* at 1 h and *Il10*/*Actb* at 3 h. Culture supernatants were analyzed to measure the production of (B) TNF-α at 4 h and (C) IL-10 at 24 h by ELISA. The data are expressed as the mean ± SD (n = 3).

The NF-κB and MAPK signaling induce the production of inflammatory cytokines that participate in inflammatory responses (Arthur and Ley, 2013; Liu et al., 2017). We investigated whether DH-I-180-3 treatment affects the levels of the proinflammatory cytokine TNF-α and the anti-inflammatory cytokine IL-10. The mRNA level of TNF-α was increased by DH-I-180-3 photoactivation in *S.* Typhimurium-infected RAW264.7 cells (Fig. 4A). However, there was no significant difference in the mRNA levels of IL-10 in response to DH-I-180-3 treatment (Fig. 4A). The secretion of TNF-α was increased by approximately 2-fold in *S.* Typhimurium-infected cells that were treated with DH-I-180-3 compared with the control cells (Fig. 4B), but no significant difference in IL-10 secretion was observed (Fig. 4C). These data demonstrate that DH-I-180-3 treatment of *S.* Typhimurium-infected macrophages upregulate the proinflammatory cytokine TNF-α.

### DH-I-180-3 promotes phagosome maturation in *S.* Typhimurium-infected mouse macrophages

**Fig. 5.**
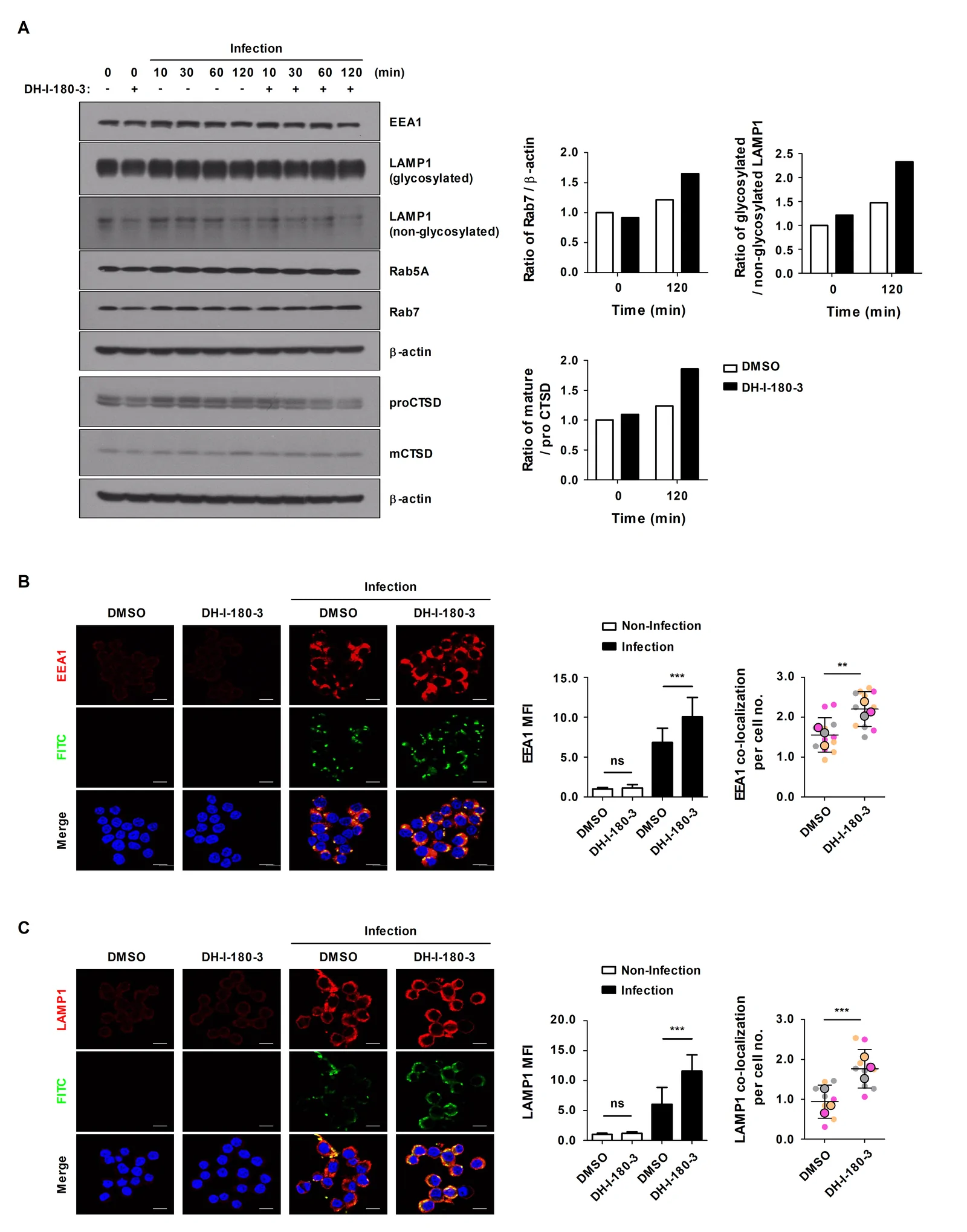
DH-I-180-3 increased the colocalization of *Salmonella* and EEA1 or LAMP1 in *S.* Typhimurium-infected macrophages. (A–C) RAW264.7 cells were pretreated with photoactivated DH-I-180-3 and then infected with *S.* Typhimurium. (A) Marker protein levels at various stages of phagosome maturation were examined by western blotting. β-Actin was used as the loading control. The bar graph shows the densitometric quantification of the bands corresponding to Rab7/β-actin, glycosylated LAMP1/nonglycosylated LAMP1, and mature CTSD (mCTSD)/proCTSD 2 h after *S.* Typhimurium infection. The data were normalized to those of noninfected and untreated cells. One representative result of three independent experiments is shown. RAW264.7 cells were fixed and stained with anti-*Salmonella* LPS-FITC and anti-EEA1-Alexa Fluor 647 (B) or anti-LAMP1-Alexa Fluor 647 antibodies (C). The left bar graph presents the relative levels of EEA1 (B) or LAMP1 (C) calculated according to the red mean fluorescence intensity. The right bar graph presents the number of puncta per cell representing EEA1^+^ (B) or LAMP1^+^ (C) phagosomes containing FITC-labeled *S.* Typhimurium. Scale bar = 10 μm. The data are expressed as the mean ± SD (n = 3 independent experiments with 50 cells per condition).

Through phagocytosis, macrophages internalize particles, microbes, and apoptotic cell debris via phagosomes, and then, phagosomes are degraded during their maturation (Jain et al., 2019). Phagosome maturation progresses through early phagosome, late phagosome, and phagolysosome stages, and representative markers corresponding to each stage are known (Lee et al., 2020c). To determine the effects of DH-I-180-3 on phagosome maturation, we compared the protein expression levels of Rab5A and EEA1 (early phagosome markers), Rab7 and LAMP1 (late phagosome markers), and Cathepsin D (CTSD, lysosome marker). The protein expression levels of the phagosome maturation markers were increased in the *S.* Typhimurium infection group (Fig. 5A). In particular, the expression levels of Rab7, glycosylated LAMP1, and cleaved CTSD tended to be increased in DH-I-180-3-treated RAW264.7 cells compared with control RAW264.7 cells at 2 h after infection (Fig. 5A). Moreover, fluorescence microscopy revealed increased accumulation of EEA1 or LAMP1 at 30 min or 2 h after infection, and this result was consistent with the result observed after DH-I-180-3 treatment (Fig. 5B and 5C). In addition, the colocalization of EEA1 or LAMP1 with *S.* Typhimurium increased by approximately 1.5-fold and 2-fold, respectively, after DH-I-180-3 treatment (Fig. 5B and 5C). These results indicate that DH-I-180-3 photoactivation promotes overall phagosome maturation.

## Discussion

The misuse and overuse of antimicrobials greatly contribute to the development of drug-resistance in pathogens; thus, it is crucial to pursue alternative therapies to clear resistant bacteria and inhibit the development of resistance (de Freitas et al., 2018). One such alternative therapy is PDT based on aPDT (Galstyan, 2021). Previous studies have reported that pheophorbide a, which is a precursor of the photosensitizer DH-I-180-3, has anticancer and antimicrobial activities as well as anti-inflammatory and immunostimulatory activities (Saide et al., 2020). These findings indicate that pheophorbide a can be applied in the treatment of various human pathologies. In our study, we confirmed a reduced number of intracellular *Salmonella* upon treatment with DH-I-180-3, but we did not observe direct antibacterial activity. This finding differs from the previously demonstrated effect of DH-I-180-3 on controlling multidrug-resistant *Mycobacterium tuberculosis* (Sung et al., 2013). Therefore, our data demonstrates that the photosensitizer DH-I-180-3 exerts antibacterial effects via a previously unexplained mechanism rather than through a conventional aPDT mechanism.

Immunostimulation is a well-established mechanism underlying the antitumor effects of PDT. The oxidative stress that is induced by PDT in tumor tissues promotes cancer cell death, induces acute inflammatory responses, and mediates antitumor immunity through secondary inflammatory mediators (Sai et al., 2021; Thomas-Moore et al., 2022). However, the immunological effects of PDT in the context of bacterial infections are poorly understood. Tanaka et al. reported that the innate immune response was stimulated by PDT in an in vivo mouse model of bacterial arthritis (Tanaka et al., 2012). Some studies have reported that PDT mediates bacterial clearance and wound healing through anti-inflammatory responses in bacterial infection models (Wang et al., 2017, 2022; Yin et al., 2022). In this study, we treated *S.* Typhimurium-infected macrophages with DH-I-180-3 and found that DH-I-180-3 activated the NF-κB and MAPK signaling pathways, leading to increased TNF-α levels.

Internalized *Salmonella* form *Salmonella*-containing vacuole (SCV) structures and prevent phagosome‒lysosome fusion through effector proteins. These SCVs serve as replicative compartments (Steele-Mortimer, 2008). We confirmed that the expression of phagosome maturation markers was upregulated during *S.* Typhimurium infection, but no notable activity of the lysosomal protease CTSD was observed in late stages of infection. However, compared with the control treatment, the DH-I-180-3 treatment markedly activated CTSD at 2 h after infection. These results suggest that DH-I-180-3 can reverse not only the early and late stages of phagosome maturation but also the lysosomal fusion avoidance mechanism of *S.* Typhimurium.

Recent studies have suggested that exposure of bacteria to ROS can enhance the emergence of multidrug resistance (Li et al., 2020b; Muehler et al., 2020). Therefore, novel antimicrobial strategies that induce ROS production are needed. In our study, we observed an increase in intracellular ROS levels in macrophages after DH-I-180-3 treatment, which was also evident during *S.* Typhimurium infection. Intracellular ROS not only contribute to the direct killing of bacteria within macrophages but also modulate the oxidative reduction of immune signaling molecules, inflammasome activation, proinflammatory cytokine production, and phagosome maturation (Herb and Schramm, 2021; Shekhova, 2020). Consequently, we propose that the DH-I-180-3-induced increase in ROS promotes intracellular inflammatory signaling and the phagosome maturation pathways in host cells, thereby regulating bacterial growth. Additionally, further research is needed to explore the direct impact of the photoactivated DH-I-180-3-induced increase in ROS levels on the intracellular bacterial clearance mechanism in macrophages.

In conclusion, our study demonstrated that the photosensitizer DH-I-180-3 stimulates inflammatory signaling pathways and promotes phagosome maturation in *S.* Typhimurium-infected macrophages, leading to the regulation of intracellular bacterial growth. Furthermore, these findings suggest that DH-I-180-3, by activating the immune response in host cells rather than exerting conventional antibacterial effects, has the potential to be utilized as a therapeutic agent for Gram-negative bacterial infections.
